# The Role of Brincidofovir in Preparation for a Potential Smallpox Outbreak

**DOI:** 10.3390/v9110320

**Published:** 2017-10-30

**Authors:** Scott A. Foster, Scott Parker, Randall Lanier

**Affiliations:** 1 Chimerix, Durham, NC 27713, USA; rlanier@chimerix.com; 2 Department of Molecular Microbiology and Immunology, Saint Louis University School of Medicine, St. Louis, MO 63104, USA; scott9379@gmail.com

**Keywords:** smallpox, variola virus, bioterrorism, bioweapon, brincidofovir, CMX001, antiviral

## Abstract

Smallpox (variola) virus is considered a Category A bioterrorism agent due to its ability to spread rapidly and the high morbidity and mortality rates associated with infection. Current recommendations recognize the importance of oral antivirals and call for having at least two smallpox antivirals with different mechanisms of action available in the event of a smallpox outbreak. Multiple antivirals are recommended due in large part to the propensity of viruses to become resistant to antiviral therapy, especially monotherapy. Advances in synthetic biology heighten concerns that a bioterror attack with variola would utilize engineered resistance to antivirals and potentially vaccines. Brincidofovir, an oral antiviral in late stage development, has proven effective against orthopoxviruses in vitro and in vivo, has a different mechanism of action from tecovirimat (the only oral smallpox antiviral currently in the US Strategic National Stockpile), and has a resistance profile that reduces concerns in the scenario of a bioterror attack using genetically engineered smallpox. Given the devastating potential of smallpox as a bioweapon, preparation of a multi-pronged defense that accounts for the most obvious bioengineering possibilities is strategically imperative.

## 1. The Need for Smallpox Antivirals

Despite official eradication in 1980, smallpox is still viewed as a significant threat due to its potential as a biological weapon [1]. The current confirmed stocks of variola virus, the etiologic agent of smallpox, are maintained at two World Health Organization sanctioned laboratories: in the United States at the Centers for Disease Control and Prevention in Atlanta, Georgia, and in Russia at the VECTOR Institute in Koltsovo, Novosibirsk Region. However, given the ubiquity of smallpox prior to its eradication, it seems likely that stocks of variola virus exist outside of these labs. Indeed, a US administration intelligence review concluded that four nations, Iraq and North Korea among them, held undeclared stocks of variola virus [2]. According to Ken Alibek, a former Soviet Union official who defected to the US, tons of highly lethal weaponized variola virus were created under a Soviet era bioweapons program [3]. In 2016, Canadian researchers formally demonstrated that synthetic biology techniques could be used to recreate an orthopoxvirus when they produced live horsepox virus from synthetic DNA obtained by mail order [4]. The ability to create replication-competent orthopoxviruses (including variola) from synthetic DNA brings additional concerns in that it would be relatively simple to modify the virus to increase infectivity or lethality, or to include resistance to antivirals and potentially vaccines.

The US Institute of Medicine concluded that antiviral agents with oral bioavailability would be important for containing the spread of smallpox in an immunologically naive population and that at least two therapeutics with different mechanisms of action should be available due to the potential for development of viral resistance [5]. Currently, tecovirimat and cidofovir are stockpiled in the US; however, cidofovir requires intravenous infusion, which would limit its utility in a widespread outbreak.

## 2. Experience with BCV

Brincidofovir (BCV, CMX001, HDP-CDV) is comprised of a lipid moiety, 3-hexadecyloxy-1-propanol (HDP), conjugated to the phosphonate of cidofovir (CDV), an acyclic nucleotide analog of deoxycytidine monophosphate. BCV retains the broad-spectrum activity of CDV against dsDNA viruses, while lipid conjugation counters two major limitations of CDV, namely nephrotoxicity and the lack of oral bioavailability [6,7]. Improvements in oral bioavailability and antiviral potency are attributable to the more efficient cellular uptake of BCV facilitated by the lipid moiety. Once inside cells, the lipid ester linkage of BCV is cleaved to liberate CDV, which is then phosphorylated to produce cidofovir diphosphate (CDV-PP). CDV-PP inhibits orthopoxvirus replication by inhibiting viral DNA polymerase-mediated synthesis of viral DNA [8].

In vitro studies have demonstrated that BCV inhibits orthopoxvirus replication, including variola virus, with enhanced potency compared to CDV [7,9]. The 50% effective concentration (EC_50_) for BCV against variola virus was estimated to be in the range of 0.05–0.21 μM, with an average of 0.11 μM across five strains chosen to represent distinct DNA polymerase genotypes [9]. The EC_50_ of BCV against rabbitpox and mousepox (ectromelia) viruses is approximately 0.5 μM; the EC_50_ against other orthopoxviruses has been reported to be in the range of 0.2–1.2 μM [6,10,11].

Initial studies of BCV efficacy in the rabbitpox model of orthopoxvirus infection were performed in the laboratory of Richard Moyer at the University of Florida [12,13]. Rabbits infected with rabbitpox virus present with a similar pattern of clinical disease as humans with smallpox, with a period of asymptomatic infection followed by fever, skin lesions, and progressive organ dysfunction; the key differentiator is that the course of rabbitpox infection is more rapid than that of human smallpox by at least two-fold. BCV efficacy was subsequently demonstrated in randomized, blinded, placebo-controlled studies in the rabbitpox model, first at the University of Florida [14] and then at a contract research laboratory (Battelle Biomedical Research Center, West Jefferson, OH, USA) [15]. A pivotal study has been completed in compliance with Good Laboratory Practice (GLP) regulations [16]. In each study, a statistically significant survival benefit was demonstrated when treatment was initiated at the onset of clinical signs of disease in the rabbits, i.e., after detection of lesions or, in the GLP study at up to 48 h after the onset of fever [15,16]. In the GLP study, rabbits treated with BCV immediately upon the development of fever had 100% survival; 93% of those treated 24 h or 48 h after the onset of fever survived. The survival advantage was statistically significant for these three arms when compared to a survival rate of 48% in the placebo group [16].

**Figure 1:**
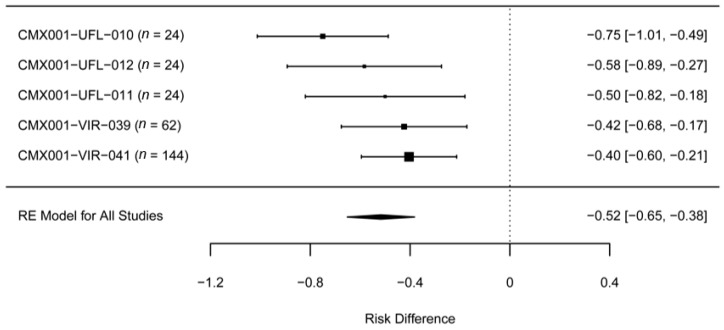
Forest plot of BCV efficacy data in the rabbitpox model. The vertical dotted line is a reference for no risk difference (i.e., no drug effect). All studies included equal numbers of male and female rabbits. Studies CMX001-UFL-010, -011, and -012 were conducted at the University of Florida [14]. In these studies, BCV was administered orally at 20 mg/kg beginning at the first observation of lesions. In Study UFL-010, animals received a total of three doses of 20 mg/kg, one dose every 48 h (20/20/20 mg/kg q48h), while in Studies UFL-011 and -012, the animals received 1 or 2 doses, respectively. Studies CMX001-VIR-039 and -041 were conducted at Battelle [15,16]. In Study VIR-039, BCV was administered at the first observation of lesions. Animals received 5/5/5, 20/5/5, or 20/20/20 mg/kg q48h. In the pivotal study VIR-041, BCV was administered at the first observation of fever, or was delayed by 24, 48, or 72 h after fever. Animals received 20/5/5 mg/kg q48h. Treatment effect was estimated by calculating the risk difference for each study. A pooled estimate of the risk difference across all studies was estimated using meta-analysis methodology. For calculation of the risk difference in each study (right column (95% CI)), the treatment groups were combined into one group of BCV-treated animals, regardless of the level of efficacy in each dose group. The pooled estimate of the risk difference is 52% (95% CI: 65% to 38%), meaning the average mortality in BCV-treated animals is 52% lower on an absolute basis. Removing BCV doses above those used in Study CMX001-VIR-041 (20/5/5 mg/kg q48 h) from the analysis (i.e., 20/20/20 mg/kg q48h and 20/20 mg/kg q48h) shifts the pooled estimate of the risk difference to 41% (95% CI: 55% to 27%).

Figure 1 displays a forest plot of the study specific risk difference estimates and corresponding 95% confidence intervals (CI) for the key BCV efficacy studies in the rabbitpox model. For calculation of the risk difference in each study, the treatment groups were combined into one group of BCV-treated animals per study, regardless of the level of efficacy in each dose group. In this regard, the risk differences may be underestimated due to the inclusion of groups with less efficacious BCV regimens or delayed BCV dosing. Specifically, in Study CMX001-VIR-039, the risk estimate includes a lower BCV dose group, and in Study CMX001-VIR-041, the risk estimate includes the 72 h delayed treatment group.

A predicted effective dose for the treatment of smallpox in humans was established by comparing the pharmacokinetic (PK) exposure of BCV in rabbit plasma to the PK of BCV in human plasma. At a dose of 200 mg of BCV once weekly for 3 weeks, the mean exposure in humans exceeds that observed at the efficacious dose in rabbits [15,16]. Furthermore, mean PK exposure of the active BCV metabolite, CDV-PP, in peripheral blood mononuclear cells (PBMCs) in humans was equal to or exceeded the PK exposure of CDV-PP in rabbit PBMCs. Based on these observations, 200 mg of BCV administered at Days 0, 7, and 14 in adults is predicted to provide an efficacious exposure of BCV for the treatment of smallpox.

**Figure 2:**
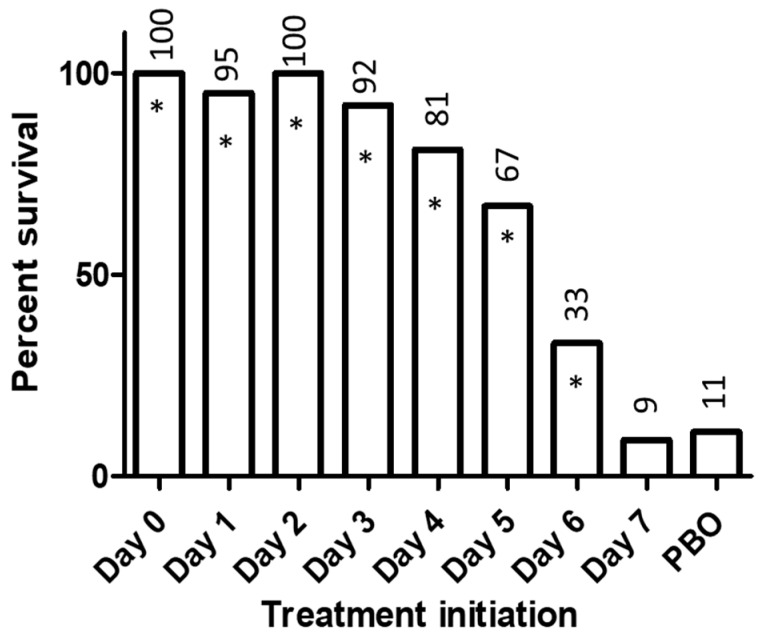
Survival by day of treatment initiation in the intranasal inoculation mousepox model. Data were pooled across studies and include different mouse strains, viral inoculum levels, and BCV dosage regimens. Data included for Studies CMX001-SLU-005, -006, -007, -008, -009, -011, -012, -013, -014, and CMX001-VIR-102 (initial BCV doses of 10 mg/kg to 30 mg/kg or placebo, single ± maintenance doses, treatment initiation Day 0 to Day 7, A Strain (A/NCR, A/J), SKH-1, C57Bl/6, and BALB/c mice). Individual studies included either male or female mice. All SKH-1, C57Bl/6, and BALB/c mice represented in the figure were female (*N* = 132, 110, and 55, respectively). A Strain mice included both males and females (*N* = 266 and 179, respectively). The numbers above the bars indicate the percentage survival. The number of animals for each treatment initiation day: Day 0 = 35, Day 1 = 20, Day 2 = 30, Day 3 = 75, Day 4 = 115, Day 5 = 144, Day 6 = 168, Day 7 = 44, PBO = 111. * Two-sided Fisher’s exact test, *p* < 0.0001 vs. placebo. No adjustments were made for multiple comparisons. These results have been previously published [17,18,19], with the exception of the BALB/c results (Study CMX001-VIR-102).

Efficacy of BCV in a second animal model, the mousepox (ectromelia virus) model of orthopoxvirus infection, has been established across numerous studies in the laboratory of Mark Buller at Saint Louis University exploring different mouse strains, viral inoculums, and BCV dosing regimens [17,18,19]. A statistically significant survival benefit was demonstrated when treatment was started as late as 6 days post-inoculation with ectromelia virus when the mean day of death of the placebo control animals was 11.3 in the same experiment [19]. Figure 2 shows a pooled analysis across several BCV efficacy studies in the mousepox model, illustrating the survival benefit based on the timing of treatment initiation. Thus, mousepox offers a second model of BCV efficacy in which treatment initiated up to the midpoint of the disease demonstrates a survival benefit.

Data from over 1400 human subjects have contributed to the BCV safety database, which includes over 1000 complex immunocompromised adult and pediatric patients, many of whom participated in studies with a nominal 12-week treatment period. Over 500 of these immunocompromised individuals received a minimum of 3 weeks of BCV at the anticipated treatment regimen for smallpox, i.e., a total weekly dose of 200 mg administered orally for 3 consecutive weeks (or 4 mg/kg in individuals with a body weight <50 kg) [20]. The majority of BCV associated adverse events reported during the first 3 weeks of treatment were gastrointestinal in nature or consisted of asymptomatic elevations in serum transaminases; these were typically mild to moderate in intensity and were transient, with no lasting effects [20].

Recognizing the available data that indicate efficacy against orthopoxviruses, BCV has been made available for use under emergency investigational new drug applications in the US and under equivalent regulations outside of the US for the treatment of progressive vaccinia [21] and cowpox [22]. In the progressive vaccinia case, BCV (6 weekly doses totaling 700 mg) was added to the regimen after numerous doses of vaccinia immune globulin and tecovirimat. While the complicated treatment of the patient makes assessment of the contribution of any single intervention difficult, tecovirimat-resistant virus was detected in the progressive vaccinia patient [21], which suggests that BCV was an important component of the patient’s treatment and ultimate survival.

Because BCV has activity against other DNA viruses, it has multiple potential uses [6,7] and has been used to treat patients infected with a variety of non-poxvirus DNA viruses, including cytomegalovirus and other herpesviruses, adenoviruses, BK virus (human polyomavirus 1), and papillomavirus.

## 3. Drug-Resistant and Enhanced Virulence Viruses

When considering the potential clinical impact of resistance on antiviral efficacy and durability, it is important to recognize the concept of “barrier to resistance”. Barrier to resistance relates to the relative ease with which viruses can become resistant to an active antiviral and encompasses numerous factors, including the genetic barrier to resistance, PK coverage, and viral fitness [23]. The genetic barrier is defined as the number of primary mutations needed for antiviral drug resistance to emerge: the more mutations required, the higher the genetic barrier [23]. PK coverage relates to the fold change in phenotypic sensitivity conferred by specific mutations and the ability or inability of attainable drug exposures to overcome whatever level of resistance is under consideration. The concept of viral fitness relates to “the relative ability to produce stable infectious progeny in a given environment”, i.e., the better a virus can replicate, the greater its fitness [24]. In order for drug resistance to be manifested in the context of antiviral treatment, the virus must acquire mutation(s) that confer resistance sufficient to overcome the available drug concentration, and the mutant virus must be able to replicate and retain virulence.

Orthopoxviruses resistant to antivirals (e.g., BCV/CDV or tecovirimat) can be selected in cell culture experiments. Numerous studies with orthopoxviruses have been conducted with CDV to investigate the development of resistance, which are relevant to BCV due to the shared active metabolite, CDV-PP. BCV/CDV resistance-associated mutations occur in the viral DNA polymerase gene. Single amino acid substitutions generally result in resistance of <10-fold (e.g., 10× higher drug concentration is needed to impact viral replication), whereas two or more substitutions can result in resistance of up to 30-fold [25]. However, in all cases tested, BCV/CDV-resistant viruses have attenuated virulence in mice [25,26]. The most common sites of BCV/CDV resistance-associated mutations are at DNA polymerase amino acid positions A314 and A684. Vaccinia virus containing the A314T and A684V displayed attenuated virulence in mice; even infection of mice with an elevated inoculum produced limited mortality (20%) and weight loss. Importantly, treatment of these mice with CDV still provided protection [26]. These observations argue that BCV has a high barrier to resistance because (1) multiple mutations are needed for high level resistance; (2) the resulting viruses have reduced virulence; and (3) attainable drug concentrations may retain efficacy against the resistant virus in vivo.

Consistent with its mechanism of action, tecovirimat resistance-associated mutations occur in the F13L gene, which is involved in the production of enveloped orthopoxvirus virions. A single mutation of G277C resulted in a cowpox virus with >800-fold phenotypic resistance [27]. Other single and multiple amino acid changes in F13L that resulted in resistance have been identified in several orthopoxviruses [28]. While the virulence of these resistant viruses has not been reported in vivo, some appeared to have little or no replication defect in cell culture and produce an enveloped virus, as evidenced by the formation of comet-shaped plaques [28]. As previously noted, tecovirimat-resistant virus (up to 50-fold) was detected in swabs from a patient with progressive vaccinia following tecovirimat treatment [21]. Sequence analysis revealed two mutations in F13L from the same swabs (A290V and L315M) that increased in frequency during treatment [21]. These findings underscore the risk of resistance with a single antiviral with a low barrier to resistance, and the need for multiple anti-orthopoxvirus agents with different mechanisms of action.

In the context of smallpox and bioterrorism, resistance takes on additional importance. Since resistant viruses can be created in the lab, either by cell culture selection or by synthetic biology, bioweapons could be created that render an existing antiviral ineffective. Equally concerning is the potential creation of viruses with enhanced virulence and/or resistance to vaccines. Ectromelia virus expressing murine interleukin 4 (IL-4) displayed increased virulence, killing mouse strains that are normally resistant to the virus, as well as mice that had been previously vaccinated [29,30]. Having available several antivirals with different resistance profiles mitigates these risks, as it increases the number of mutations required for viral breakthrough and decreases the chance that the resulting virus will retain full pathogenicity. Brincidofovir’s high barrier to resistance, as well as the significant impact on replication competence and pathogenicity observed for viruses with engineered BCV/CDV resistance, provides additional protection against these synthetic viral threats.

## 4. Combination Therapy

Antiviral therapy using combinations of drugs is often more effective and durable than monotherapy. Properly designed combination therapy has the potential to improve antiviral response and reduce the risk of resistance development [31]. In HIV treatment, standard antiviral regimens evolved to include three antivirals from at least two classes, typically two nucleoside reverse transcriptase inhibitors and one integrase inhibitor, protease inhibitor, or non-nucleoside reverse transcriptase inhibitor [32,33,34,35]. Similarly, antiviral combinations are standard in treating the most prevalent genotypes of hepatitis C [36]. As expected, based on their different viral targets, the combination of tecovirimat and BCV was synergistic against orthopoxviruses in cell culture and in studies of cowpox virus infection of mice [37]. The combination of tecovirimat and BCV was also superior to either agent alone in experiments using enhanced virulence ectromelia virus expressing murine IL-4; neither tecovirimat nor BCV alone (at the doses used in the study, 100 mg/kg and 4 mg/kg, respectively, daily for 14 days) was effective in improving survival, whereas the combination effectively reduced mortality [30]. In the previously noted case of progressive vaccinia, BCV was added to the patient’s regimen following initial treatment with tecovirimat and emergence of tecovirimat resistance [21]. The authors noted that, “in such severely ill patients, combination therapy may best be initiated at the outset, which might reduce the viral load and subsequent development of antiviral resistance mutations” [21]. This proposal aligns with current therapeutic guidance for other viral diseases.

## 5. Conclusions

Given the established ability to “weaponize” variola virus and the potential for synthesis of drug-resistant or enhanced virulence variants, it is imperative our medical countermeasure strategy encompasses multiple therapeutic options, including vaccination and antivirals with different mechanisms of action and no cross-resistance. BCV has demonstrated a significant survival benefit even after treatment initiation was delayed to the mid-point of disease progression in two relevant animal models of orthopoxvirus infection. In addition, BCV is progressing in clinical development as a treatment for life-threatening viral infection in patients with impaired immune response due to transplant or congenital immunodeficiency. BCV’s mechanism of action complements that of the currently stockpiled tecovirimat and could be a critical component in an effective response to a smallpox outbreak involving drug-resistant or enhanced virulence virus. In the face of an increasing threat posed by smallpox as a bioweapon, including BCV in our readiness planning is supported by its demonstrated survival benefit in animal models, relevant clinical experience in high-risk adult and pediatric populations, robust clinical safety database, synergy with tecovirimat, and high barrier to resistance.
